# Baló’s concentric sclerosis is immunologically distinct from multiple sclerosis: results from retrospective analysis of almost 150 lumbar punctures

**DOI:** 10.1186/s12974-017-1043-y

**Published:** 2018-01-18

**Authors:** S. Jarius, C. Würthwein, J. R. Behrens, J. Wanner, J. Haas, F. Paul, B. Wildemann

**Affiliations:** 1 Molecular Neuroimmunology Group, Department of Neurology, University of Heidelberg, Heidelberg, Germany; 2 NeuroCure Clinical Research Center, Charité—Universitätsmedizin Berlin, Berlin, Germany; 3 Department of Neurology, Charité—Universitätsmedizin Berlin, Berlin, Germany; 4 Clinical and Experimental Multiple Sclerosis Research Center, Department of Neurology, Charité—Universitätsmedizin Berlin, Berlin, Germany; 5 Experimental and Clinical Research Center, Max Delbrueck Center for Molecular Medicine and Charité Universitätsmedizin Berlin, Berlin, Germany

**Keywords:** Baló’s concentric sclerosis (BCS), Baló, Multiple sclerosis (MS), Cerebrospinal fluid (CSF), Lumbar puncture, Oligoclonal bands (OCB), Link’s IgG index, Intrathecal IgG synthesis, Pleocytosis, Magnetic resonance imaging, Histopathology, Pattern III MS, Diagnosis, Immunology/immunopathology, Review of the literature

## Abstract

**Background:**

Baló’s concentric sclerosis (BCS) is a rare inflammatory demyelinating disorder of the central nervous system characterised by concentric layers of demyelination. It is unclear whether BCS is a variant of multiple sclerosis (MS) or a disease entity in its own right.

**Objective:**

To compare the cerebrospinal fluid (CSF) features of BCS to those of MS.

**Methods:**

Retrospective analysis of the CSF profile of all patients with BCS reported in the medical literature between 1980 and 2017.

**Results:**

In total, the results of 146 lumbar punctures (LP) in 132 patients were analysed. The most striking finding was a lack of CSF-restricted oligoclonal bands (OCB) in 66% (56/85) of all LP in the total BCS group, in 74% (14/19) in the subgroup of patients with both MRI and histological evidence for BCS, and in 82% (18/22) in the subgroup of patients with highest radiological confidence (high MRI quality, ≥ 3 layers of demyelination). OCB disappeared in 1/2 initially OCB-positive patients. These findings are in stark contrast to MS, in which OCB are present in ≥ 95% of patients and are thought to remain stably detectable over the entire course of disease (*p* < 0.000001). OCB frequency was low both in ‘historic’ patients (1980–2009; 37%) and in more recent patients (2010–2017; 31%). OCB-positive and OCB-negative patients did not differ significantly with regard to age, sex, disease duration, number of Baló-like lesions on MRI, number of relapses, treatment or final outcome. In accordance with the high rate of OCB negativity, Link’s IgG index was negative in 63% of all tested samples (*p* < 0.000001 vs. MS). CSF pleocytosis was present in 28% (27/96; *p* < 0.000001 vs. MS) and elevated CSF total protein levels in 41% (31/76) of samples.

**Conclusion:**

OCB and IgG index frequencies in BCS are much more similar to those reported in neuromyelitis optica or myelin oligodendrocyte glycoprotein antibody-associated encephalomyelitis than to those in MS. Our findings suggest that in most cases, BCS-like lesions denote the presence of a disease entity immunologically distinct from MS. In addition, we provide data on the demographics, clinical course and radiological features of BCS based on the largest cohort analysed to date.

## Background

Baló’s concentric sclerosis (BCS) is a rare demyelinating disorder of the central nervous system (CNS) characterised by concentric layers of demyelination as detected by MRI or histopathology, and named after the Hungarian pathologist József Baló (1895-1979) [1, 2]. BCS is often regarded as a rare variant of multiple sclerosis (MS). While there are indeed similarities in terms of clinical presentation, BCS differs in several aspects from typical MS: lesion morphology (onion-like configuration characterised by concentric, alternating layers of demyelinated and myelinated tissue), lesion size (high frequency of large or ‘tumefactive’ lesions), disease course (often monophasic and self-limiting rather than relapsing-remitting) and disease severity (not uncommonly fatal at first occurrence). This suggests that BCS and MS may be distinct disorders.

In a number of studies, we have demonstrated highly significant differences in cerebrospinal fluid (CSF) features between patients with MS and patients with other autoimmune disorders of the CNS. In particular, we have found significantly lower frequencies of CSF-restricted oligoclonal bands (OCB) and other markers of intrathecal IgG synthesis in patients with AQP4-IgG-positive neuromyelitis optica (NMO) spectrum disorders (NMOSD), myelin oligodendrocyte glycoprotein antibody-associated encephalomyelitis (MOG-EM), acute demyelinating encephalomyelitis (ADEM) and paraneoplastic neurological disorders (PND) than in MS, as well as significant differences in intrathecal IgG composition and dynamics and in blood–CSF barrier function [3–14]. This indicates that studying CSF profiles may be helpful in distinguishing clinically related but immunopathogenetically distinct diseases.

To address the question of whether BCS is a variant of multiple sclerosis (MS) or an immunologically distinct disease entity in its own right, we therefore set out to systematically compare the CSF features of BCS with those in MS.

## Methods

We performed a systematic review of all BCS cases published in English, German, French, Spanish, Japanese, Chinese, Russian or Italian in journals indexed in the PubMed database of the US Library of Science at the US National Institutes of Health between 1980 and 2017. Relevant publications were identified using the following search expression: Balo[title/abstract] OR Balo’s[title/abstract] OR Balos[title/abstract] OR ‘concentric sclerosis’[title/abstract]. Further publications/cases were identified by screening the reference lists of all PubMed-retrieved publications and from the authors’ own files. Overall, 215 publications that made mention of Baló’s concentric sclerosis in the abstract and/or title were identified and information on the CSF findings of 132 patients with BCS was collected. Results from follow-up lumbar punctures (LP) were available from 12/132 (9%) patients (10 × 2 LP, 2 × 3 LP). In total, results from 146 LP in 132 patients diagnosed with BCS were available for retrospective analysis.

The following CSF parameters were assessed: CSF-restricted oligoclonal IgG bands (OCB); Link’s IgG index ([IgG CSF/IgG serum]/[albumin CSF/albumin serum]); immunoglobulin G/A/M (IgG/A/M) CSF levels and IgG/A/M CSF/serum ratios (QIgG/A/M); measles/rubella/zoster reaction (MRZR) as defined by a positive reaction to at least two of the three viral agents; CSF albumin concentration and albumin CSF/serum ratio (QAlb); CSF white cell count (WCC); CSF cytology; CSF total protein concentration (TP); frequency of an albuminocytologic dissociation (ACD), i.e. CSF TP elevation in the absence of CSF pleocytosis; CSF myelin basic protein concentration (MBP); and CSF lactate concentration. A CSF white cell count ≥ 5/μl was classified as increased. An age-dependent upper reference range for CSF L-lactate was applied (0–15 years of age, 1.8 mmol/l; 16–50, 2.1 mmol/l; > 50, 2.6 mmol/l). The upper reference limit for total CSF protein was set at 50 mg/dl [15].

In addition, the following demographic, clinical and paraclinical parameters were assessed: sex, ethnic origin, age at onset, age at LP, time between last attack and LP, attack and long-term outcome, treatment status at the time of LP, number and location of Baló-like lesions, frequency and number of coexisting non-Baló-like lesions, initially suspected diagnosis, level of diagnostic certainty (radiological vs. radiological plus histological evidence), and outcome at last follow-up (fatal, no recovery, almost no recovery, partial recovery, almost full recovery, full recovery).

The Mann–Whitney *U* test and Fisher’s exact test (two-tailed) were used to compare groups. Spearman’s *r* was used to test for correlations between groups. Due to the exploratory nature of this study, no correction for multiple testing was performed. All analyses were performed retrospectively; accordingly, no CSF or serum samples were obtained for this study.

## Results

### Patient characteristics

The sex ratio (male to female) was 1:1.54. The median age of disease onset was 32 years (range 4–57). In 68 patients, the diagnosis of BCS was based upon typical radiological features; in 35, on typical histopathological features; and in 25, on both typical radiological and typical histopathological features. Histopathological evidence was obtained from biopsy samples in 15, from autopsy samples in 42 and from both biopsy and autopsy samples in 3 patients. In 43 cases, a single Baló-like lesion was present at the time of LP as detected by MRI or, if MRI was not available, histology, and multiple (median 2; range 2–9) Baló-like lesions were noted in 53 cases. Baló-like lesions were located in the supratentorial brain at the time of LP in 93 cases, in the infratentorial brain in 3, in both the supra- and infratentorial brain in 5 and in the supra- and/or infratentorial brain and the spinal cord in 4. Additional non-Baló-like CNS lesions were present in 58/94 (61.7%) patients at the time of LP. Before the diagnosis of BCS was made, MS was initially suspected in at least 8 patients, stroke in 5 and other diagnoses such as ‘tumefactive demyelinating disease’, ‘brain tumour’ or ‘brain abscess’ in 8 (no data in the remainder). Antibodies to aquaporin-4 (AQP4-IgG) or myelin oligodendrocyte glycoprotein (MOG-IgG) had been tested in 20 and 3 patients, respectively, and were negative in all. The median age at first LP was 32 years (range 4–60). The exact time between onset of the most recent attack and LP was given for 54 LP and was ≤ 30 days in 42 cases; the median interval was 15 days (range 1–190 days). In 58 additional cases, the exact time interval was not specified but LP was performed during an acute attack and thus probably within 30 days of attack onset. At last follow-up (median observation period 0 year; range 0–14), more than one attack had occurred in 27/102 (26.5%) patients. Fifty-eight LP were performed prior to treatment with steroids or immunosuppressants, 4 LP in patients who had been treated with steroids shortly beforehand, 1 LP in a patient treated with immunosuppressants and 1 LP in a patient who had been treated with PEX and steroids 6 months previously [16]; in the remainder, no treatment was mentioned.

### CSF findings

The most striking finding was a lack of CSF-restricted OCB in the majority of cases: 65% (52/80) of all patients were negative for OCB (N=51) or only transiently positive (N=1) (Table 1). Overall, OCB were absent in 66% (56/85) of samples. These findings are in stark contrast to MS, in which CSF-restricted OCB are present in ≥ 95% of patients, are thought to remain stable over the entire course of disease and are considered a diagnostic mainstay (*p* < 0.000001 when compared to a reference work on OCB in MS [17]). Of note, OCB disappeared later in the disease course in 1/2 initially OCB-positive patients with available follow-up data. 3/3 initially OCB-negative patients with follow-up data remained negative for OCB upon retesting.

**Table 1:** Intrathecal synthesis, cellular immune response and total protein concentrations in BCS and MS

	BCS	MS	*p* value
Number of patients	132	267 [17]	n.a.
Number of samples	146	267 [17]	n.a.
Sex ratio (male to female)	1:1.54	1:1.95 [17]	n.s.
Median age at first lumbar puncture (range)	32 years (4–60)	36 years [17]	n.d.
Relapsing disease course	27/102 (26.5%)	n.d. [17]	n.d.
CSF-restricted OCB, positive, samples	34.1% (29/85)	98.1% (262/267) [17]	< 0.000001
CSF-restricted OCB, positive, patients	35% (28/80)	98.1% (262/267) [17]	< 0.000001
CSF-restricted OCB, transiently positive, patients	50% (1/2)	n.d.	n.a.
Link’s IgG index, elevated, samples	22.6% (7/31)	86.4% (51/59) [20]	< 0.000001
Link’s IgG index, elevated, patients	25% (7/28)	86.4% (51/59) [20]	< 0.000001
CSF WCC, elevated, samples	28.1% (27/96)	58.4% (156/267) [17]	< 0.000001
CSF WCC, median and range, if increased, samples	27/μl (6–371)	< 40/μl in 98% [17]	n.a.
CSF TP, elevated, samples	40.8% (31/76)	n.d.	n.a.
CSF TP, median and range, if increased, samples	75 (46–280)	n.d.	n.a.
ACD, samples	23.6% (17/72)	1.9% (1/54)	< 0.0005

*ACD* albuminocytologic dissociation, *BCS* Baló’s concentric sclerosis, *CSF* cerebrospinal fluid, *MS* multiple sclerosis, *n.a.* not applicable, *n.d.* no data, *n.s.* non-significant, *OCB* oligoclonal bands, *TP* total protein, *WCC* white cell count

Immunosuppressive treatment may theoretically affect OCB frequency. However, the difference between BCS patients and MS in terms of OCB positivity was still highly significant after exclusion of all patients treated with immunosuppressants (IS) at the time of LP (post hoc subgroup analysis; corrected *p* < 0.00001). Moreover, so far, an effect of IS on OCB positivity in MS has been shown only for natalizumab [18, 19], which was not used in any of the patients analysed in this study.

The difference was equally marked when only patients with both radiological and histopathological evidence for BCS and thus particularly high diagnostic certainty were considered (OCB negative in 73.7% [14/19]).

Due to the retrospective nature of the study, MRI quality varied considerably among cases. To exclude the possibility that the low rate of OCB was due to unintentional inclusion of patients with diagnoses other than BCS, we defined (in a fashion blinded to OCB results) a subgroup of cases with available high-quality MRI depicting textbook-like BCS lesions (*N* = 25); all of these lesions showed three or more layers of demyelination. In this subgroup of cases with particularly high radiological confidence, the lack of OCB was even more pronounced than in the total cohort (OCB negative in 81.8% [18/22]; OCB not tested in 3).

Finally, to rule out any chance that the low OCB rate found in our study partially reflects differences in sensitivity between current and ‘historic’ methods used to detect CSF-restricted OCB, we compared the frequency of OCB in reports published during the past 10 years with that in the older reports. However, no significant difference in OCB rates was observed between the ‘current’ and the ‘historic’ subgroup, with an even lower rate in the ‘current’ subgroup (31 vs. 37%).

We were then interested in whether OCB may define distinct subgroups of BCS patients. However, no significant differences were found between OCB-positive and OCB-negative patients with regard to sex (male to female ratio 1:1.8 vs. 1:1.4), median age at onset (32 vs. 30 years), median age at LP (32 vs. 32 years), disease course at last follow-up (relapsing in 36 vs. 34%), number of Baló-like lesions (more than one in 48.1 vs. 55.6%), location of Baló-like lesions (presence of infratentorial and/or spinal cord lesions 3.4 vs. 13.7%) and co-existing non-Baló-like lesion (present in 48.1 vs. 55.6%). While more patients in the OCB-negative group had an unfavourable outcome (fatal or no/almost no recovery in 23.3 vs. 9.5% among OCB-positive patients; *N* = 64), the differences did not reach statistical significance. Moreover, as a potentially important bias, most fatal cases were reported in Chinese, Philippine and Japanese patients in the 1980s and early 1990s, when OCB were not yet generally determined in those countries. However, if only cases published during the past 20 years (1998 to 2017) were considered, again, more OCB-negative than OCB-positive patients had an unfavourable outcome (fatal or no/almost no recovery in 20.5 vs. 7.1%; *N* = 53; *p*=n.s.).

Conversely, no statistically significant differences in OCB frequency were found between male and female patients (31.3 vs. 37.5%), between children/juveniles and adults (50 vs. 32.9%), between patients with a relapsing and patients with a non-relapsing disease course at last follow-up (36 vs 34%), between LP in patients with a single Baló-like lesion and in those with multiple Baló-like lesions (36.8 vs 32.4%) or between LP in patients with additional, non-Baló-like lesions and in those without such lesions (36.4 vs 27.6%). A non-significant trend towards a lower frequency of OCB in patients with no/almost no recovery or fatal outcome compared with patients who had partial to full recovery at last follow-up was noted (20 vs. 37.3%; *p*=n.s.).

In accordance with the widespread lack of IgG OCB, Link’s IgG index was positive only in 22.6% (7/31) of all samples tested for that parameter. This is in contrast to the reference study in MS, which reported an elevated IgG index in 86.4% (51/59; *p* < 0.000001) [20].

The CSF WCC was elevated in 27/96 (28.1%) samples. This compares to a CSF pleocytosis rate in MS of 58.4% reported in a reference work on CSF in MS (*N* = 267; *p* < 0.000001) [17]. Among patients with pleocytosis and available data, the median WCC was 27/μl (quartile range 14.75–32.75). The frequency of OCB was higher in samples with pleocytosis than in samples without pleocytosis (58.8 vs. 20%); conversely, the frequency of CSF pleocytosis was lower in OCB-negative patients than in OCB-positive patients (55.6 vs. 17.9%; *p* = 0.013).

TP was elevated in 40.8% (31/76; median 75 mg/dl; quartile range 54–98.8) of all CSF samples. Most patients with elevated TP and available data were OCB-negative (77.8% or 14/18). A so-called ACD, i.e. an increase in the CSF total protein concentration without an accompanying increase in the number of CSF white cells, was found in 17/72 (23.6%) samples. ACD is typically found in Guillain-Barre syndrome but not usually in MS (ACD in 1/53 [1.9%] LPs in 43 patients with MS according to McDonald et al., 2001 [45 × RRMS, 8 × SPMS]; unpublished data, S.J.; *p* < 0.0005 vs. BCS). The majority of patients with ACD and available data (90.9% or 10/11) were OCB negative. CSF MBP concentration was determined in seven samples and was elevated in three (42.9%).

Data on OCB pattern, IgG/A/M CSF and serum concentrations and/or QIgG, albumin CSF and serum concentrations, lactate CSF concentrations and CSF/serum antibody indices for measles, rubella or varicella zoster virus (MRZ reaction) were reported only in single patients or not at all, preventing systematic analysis of these CSF parameters.

43.2% (35/81) of all CSF examinations showed neither pleocytosis nor elevated TP, and 15.1% (11/73) showed neither CSF pleocytosis or TP elevation nor intrathecal IgG synthesis. Of note, 12 further LP were classified as ‘normal’ by the authors, but no more detailed information was provided. While these LP could not be included in the statistical analysis, they suggest that the low rate of WCC pleocytosis, OCB positivity and IgG index elevation observed in our study may even underestimate the real lack in CSF abnormalities in BCS and, in consequence, the real degree of difference between BCS and MS in terms of CSF pathology.

## Discussion

Traditionally, BCS is considered a variant of MS. Here, we demonstrate highly significant differences in CSF profiles between patients with BCS and patients with MS. In particular, we found that CSF-restricted OCB, a qualitative marker of intrathecal IgG synthesis, are missing in almost two-thirds of patients with BCS (*p* < 0.000001 compared with MS) and that Link’s IgG index, a quantitative marker of intrathecal IgG synthesis, is negative in more than 75% of cases (*p* < 0.000001 compared with MS). Given that intrathecal IgG synthesis is considered a diagnostic mainstay of MS [15, 17, 21], our findings challenge the classification of BCS as a morphological variant of MS. They rather suggest that BCS is probably caused by a disorder (or disorders) immunopathogenetically distinct from MS, at least in the majority of cases.

**Fig. 1:**
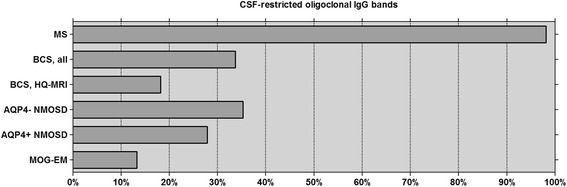
Frequency of CSF-restricted OCB in MS (*N* = 276) [17], BCS (present study; *N* = 146), NMOSD (*N* = 144) [4] and MOG-EM (*N* = 45) [11]. AQP4+ aquaporin-4-IgG-positive, AQP4− aquaporin-4-IgG-negative, BCS Baló’s concentric sclerosis, HQ-MRI high-quality MRI subgroup (see the ‘Results’ section for details), MOG-EM myelin oligodendrocyte glycoprotein antibody-positive encephalomyelitis, MS multiple sclerosis, NMOSD neuromyelitis optica spectrum disorders

A very low prevalence of CSF-restricted OCB has also been found in other diseases that were previously considered ‘variants’ of MS and are now recognised as immunologically distinct disease entities in their own rights by most experts, namely in AQP4-IgG-positive NMO [3, 4] and in MOG-EM [11] (Fig. 1). The latter two disorders often meet the clinico-radiological criteria for MS but differ from conventional MS in terms of immunopathogenesis and optimum treatment [11, 12, 22–29].

In addition, we found a significantly lower frequency of CSF pleocytosis in BCS than in MS reference cohorts (*p* < 0.000001), further corroborating the notion that BCS and MS are, in most cases, distinct disorders.

Other CSF parameters, such as CSF and serum levels of IgG and albumin, QIgG, QAlb (a marker of blood–CSF barrier function and CSF flow rate) and CSF lactose levels, were reported in only a few patients, preventing statistical assessment.

In the effort to better understand the significance of OCB in BCS, we looked into possible differences between OCB-positive and OCB-negative patients with BCS with regard to demographics, disease course, MRI findings and disease outcome. However, no marked differences between the two groups were found in terms of age at onset, age at the time of LP, sex ratio, disease course, lesion location or the number of Baló-like lesions on MRI. Conversely, the rate of OCB-positive samples did not differ significantly between male and female patients, relapsing and non-relapsing patients, children and adults, or patients with a single Baló-like lesion and patients with multiple Baló-like lesions. However, a non-significant trend towards an association of OCB negativity with more severe disease outcome was found.

### Why do some patients with BCS exhibit OCB while others do not?

We first hypothesised that the presence or absence of intrathecal IgG synthesis might depend on disease activity, as has been demonstrated in AQP4-IgG-positive NMOSD [3]. Interestingly, OCB indeed disappeared over time in one of the only two OCB-positive BCS patients with available follow-up data on OCB. However, OCB positivity rates in samples taken within 1 month after disease onset and samples taken later in the course of disease did not differ significantly in this study, arguing prima facie against a major role of disease activity.

Alternatively, BCS lesions may not denote a single disease entity but may be a (optional or non-optional) final pathway of various demyelinating diseases, e.g. reflecting the presence of intralesional hypoxia as recently proposed. If so, BCS lesions could occur in the context of both conventional (OCB-positive) MS and other (mostly or exclusively OCB-negative) inflammatory demyelinating conditions such as MOG encephalomyelitis, ADEM, NMOSD or other as yet unidentified disorders. In potential keeping with that hypothesis, there was substantial heterogeneity among BCS cases in terms of disease course (relapsing vs. monophasic), MRI presentation (additional non-BCS lesions vs. no additional lesions) and outcome (full recovery vs. fatal). Moreover, some patients with BCS had initially been diagnosed with MS, ADEM or NMOSD. Finally, at least some patients with BCS may suffer from what has been called ‘pattern III’ MS. ‘Pattern III’ MS is diagnosed on the basis of histopathological features, some of which suggest ischemia-mediated tissue damage, and has only recently been proposed to denote a disease entity distinct from classical MS based on our observation that patients with ‘pattern III’ lesions are typically negative for OCB [9]. While probably overrepresented in biopsy/autopsy samples, ‘pattern III MS’ must be a rare disease in the general MS population given that OCB are highly frequent in MS. So far, lesions showing the typical histopathological characteristics of ‘pattern III’ lesions have been reported in 12 patients with ‘concentric demyelination’ [30].

Interestingly, a recent study reported a loss of aquaporin-4 (AQP4) and other astrocytic proteins in BCS, which in some lesions preceded the loss of myelin, suggesting that BCS might, at least in a subset of cases, be primarily an astrocytopathy, just as AQP4-IgG-positive NMO [31]. However, IgG or complement deposits, as seen in NMO, are usually absent in BCS lesions, and only a single NMO-IgG-positive patients with BCS lesions has been described so far [32].

Our study has both strengths and limitations. Among its strengths, we count the very high number of LP examined (given the very low prevalence of the disease) and the fact that our results were both highly significant and robust when retested in subgroups with particularly high diagnostic certainty (see the ‘Results’ section for details). With no differences between historic and more current patients in terms of OCB frequency and only very few patients having been treated at the time of LP, we can also broadly rule out an effect of assay sensitivity or treatment; moreover, the very high rate of OCB in classic MS is not a new finding linked to modern techniques but was reported in studies performed as early as some of the earliest BCS studies analysed here.

On the other hand, one may count the retrospective nature of our study and the fact that the patients included in the analysis were seen at many different centres among the potential limitations. However, it is virtually impossible to perform a large prospective single-centre study on BCS given the condition’s very low prevalence. Moreover, the study’s retrospective ‘multicentre’ setting reduced potential risks resulting from centre-specific selection bias, a problem inherent to single-centre studies. Second, no CSF results were given in numerous reports on BCS in the previous literature. This might have introduced a bias towards cases with pathological CSF findings. However, this would imply that our results actually underestimate the observed lack of OCB, IgG index elevation and WCC pleocytosis in BCS and would thus make our findings even more relevant.

The present study focused on immunological parameters routinely tested in patients with MS and BCS. Further immunological studies aiming at investigating the differences between BCS and MS in terms of immunopathogenesis in greater detail are now required.

It is reassuring that our results are in agreement with a previous much smaller study performed on behalf of the MAGNIMS network [33]. Following another approach and focusing on MRI findings, the authors identified 69 adult patients with ‘atypical idiopathic inflammatory demyelinating lesions’ and high-quality MRI data in the English-language literature published between 1984 and 2004 and retrospectively classified 11 of them as having BCS based on typical MRI. Of those 11 BCS patients, only 9%, i.e. one patient, had OCB.

## Conclusion

In summary, our study demonstrates significant differences in CSF findings between patients with BCS and patients with MS, especially in terms of intrathecal IgG synthesis. This suggests differences in immunopathogenesis between BCS and MS and supports the notion of Baló-like lesions denoting the presence of a disorder immunologically distinct from MS in most cases. This could have clinically important diagnostic, nosological, prognostic and therapeutic implications. Of note, the OCB frequency in BCS is more similar to that reported in NMOSD and MOG-EM than in classic MS (Fig. 1). Further studies are now warranted to confirm these findings in a prospective manner as well as to look into potential differences between OCB-negative and OCB-positive patients with BCS in more detail. Given the rarity of the condition, this will require an international multicentre approach. Our results provide the rationale for such studies.

## Abbreviations

AQP4: Aquaporin-4
BCS: Baló’s concentric sclerosis
CNS: Central nervous system
CSF: Cerebrospinal fluid
EM: Encephalomyelitis
IgA: Immunoglobulin A
IgG: Immunoglobulin G
IgM: Immunoglobulin M
MOG: Myelin oligodendrocyte glocyoprotein
MRI: Magnetic resonance imaging
MRZR: Measles/rubella/zoster reaction
MS: Multiple sclerosis
NMO: Neuromyelitis optica
NMOSD: NMO spectrum disorders
OCB: Oligoclonal bands
QAlb: Albumin CSF/serum ratio
QIgG/A/M: IgG/A/M CSF/serum ratio
TP: Total protein
WCC: White cell count
